# Decoding Viral Dark Matter: Metagenomic Prokaryotic Virus Characterization With Pharokka, Phold, and Phynteny

**DOI:** 10.1002/cpz1.70405

**Published:** 2026-07-07

**Authors:** George Bouras, Susanna R. Grigson, Lindsay Durr, Bhavya Papudeshi, Vijini Mallawaarachchi, Sarah Vreugde, Robert A. Edwards

**Affiliations:** ^1^ School of Medicine, College of Health Adelaide University Adelaide Australia; ^2^ The Department of Surgery – Otolaryngology Head and Neck Surgery Central Adelaide Local Health Network Adelaide Australia; ^3^ DOE Joint Genome Institute Lawrence Berkeley National Laboratory Berkeley California; ^4^ Department of Fundamental Microbiology University of Lausanne Lausanne Switzerland; ^5^ Flinders Accelerator for Microbiome Exploration, College of Science and Engineering Flinders University Bedford Park Australia

**Keywords:** bacteriophage, genome annotation, metagenomes, prokaryotic viruses, protein function, protein structure

## Abstract

Viral metagenomics is an increasingly powerful tool for understanding the function and structure of viruses across the diverse environments of our planet. However, decoding the functional potential of prokaryotic viral metagenomes is extremely challenging. Pharokka, Phold, and Phynteny are complementary open‐source prokaryotic viral genome annotation tools that utilize a variety of bioinformatics approaches to maximally annotate viral metagenomes. This article describes a protocol for installing and running these tools on a viral metagenomic dataset, followed by visualization of annotations using our client‐side Phold Plot web assembly application. © 2026 The Author(s). *Current Protocols* published by Wiley Periodicals LLC.

**Basic Protocol 1**: Prokaryotic viral metagenome annotation with Pharokka

**Basic Protocol 2**: Enhanced prokaryotic viral metagenome protein annotation using protein structures with Phold

**Basic Protocol 3**: Further prokaryotic viral metagenome protein annotation using genome synteny and protein language models with Phynteny

**Basic Protocol 4**: Visualization of prokaryotic viral metagenome annotations with Phold Plot web assembly application

## INTRODUCTION

Viruses, especially bacteriophages (viruses that infect bacteria), are the most prevalent biological entity on the planet (Clokie et al., 2011). The advances in sequencing methodologies, reduced sequencing costs, and improvements in metagenomic assembly algorithms have exponentially increased the number of viral metagenome‐assembled genomes (vMAGs) recovered (Edwards & Rohwer, 2005; Mallawaarachchi et al., 2023, 2024). Characterizing these genomes is important for applications including phage therapy against antibiotic‐resistant bacteria (Pirnay et al., 2024), understanding microbial ecosystem dynamics (Roux et al., 2016), and uncovering novel proteins and enzymes with biotechnological potential (Al‐Shayeb et al., 2020). However, the functional potential of viral genomes remains difficult to decode due to their rapid evolution and extreme genomic diversity (Cook et al., 2024). In particular, many viral genes lack detectable homologs in existing databases, resulting in a large proportion of proteins being annotated as hypothetical or unknown, which limits biological interpretation of newly recovered genomes (Grigson, Bouras, Dutilh, et al., 2025).

This article focuses on viral metagenomic annotation using Pharokka (Bouras et al., 2023), Phold (Bouras et al., 2026) and Phynteny (Grigson, Bouras, Papudeshi et al., 2025), complementary interoperable phage genome annotation pipelines built using Python. Pharokka conducts gene calling [including identifying coding sequences (CDS), transfer RNAs (tRNAs), transfer‐messenger RNAs (tmRNAs), and CRISPR repeats] followed by sequence homology–based protein functional annotation with MMseqs2 (Steinegger & Söding, 2017) and PyHMMER (Finn et al., 2011; Larralde & Zeller, 2023). Using the output of Pharokka, Phold then uses the protein language model ProstT5 (Heinzinger et al., 2024) to infer Foldseek 3Di tokens (van Kempen et al., 2024) that represent each protein's structure, which is searched against a database of >1.36 million phage protein structures with functional labels using Foldseek. This allows annotation of viral dark matter that is extremely divergent in sequence from known proteins. Phynteny then uses genome synteny combined with the ESM2 (Lin et al., 2023) protein language model embeddings and hybrid cross attention to annotate remaining unknown proteins. Additionally, plotting capabilities, available in a user‐friendly form via the Phold Plot web assembly application, allow for easy visualization of annotated genomic features.

Combined, Pharokka, Phold, and Phynteny provide genome annotation that can quickly, routinely, and comprehensively identify the functional capability across metagenomic prokaryotic viruses of interest. Pharokka, Phold, and Phynteny Transformer are easy to install, with dependency management handled via Bioconda. Detailed user manuals describing installation, set up, and running the tools are presented via the README page of their respective GitHub repositories (https://github.com/gbouras13/pharokka; https://github.com/gbouras13/phold; and https://github.com/susiegriggo/Phynteny_transformer) and in dedicated documentation pages.

## Basic Protocol 1 PROKARYOTIC VIRAL METAGENOME ANNOTATION WITH PHAROKKA

The aim of this protocol is to use sequence homology‐based methods for initial prokaryotic viral metagenome annotation using Pharokka. The protocol involves a number of preparatory steps before running Pharokka, such as the installation of conda (via Miniforge) and the creation of a dedicated Pharokka conda environment to ensure Pharokka runs smoothly. It concludes by showing how to optionally run Pharokka with a custom HMM database for extra user‐defined annotations.

### Necessary Resources

#### Hardware

A computer with a minimum of 1 CPU core, 8 gigabytes (GB) of random‐access memory (RAM), and 5 GB of free disc storage for the database install
  Our tests using the example dataset revealed that leveraging 8 CPU cores operating at base clock speeds of 2.4 GHz and using 16 GB of RAM enabled the completion of Pharokka in ∼3 min.

##### Software

Linux (or emulators such as Windows Linux Subsystem) or MacOS operating system

##### Files

Provided example dataset
  Our test data is 63 complete metagenome assembled phage genomes from human gut samples sequenced in Cook et al. (2024) as used for benchmarking in the Phold manuscript. Each contig represents a separate viral metagenome assembled genome.

#### Download and installation of software, example data, and required databases

1 Download the test data from the Pharokka repository:


curl ‐L ‐o cook_complete_genomes.fasta https://raw.githubusercontent.com/gbouras13/pharokka/master/tests/test_data/overall/Meta_example/cook_complete_genomes.fasta

2 Install Conda. We recommend using miniforge. For more information, please follow the installation instructions at: https://github.com/conda‐forge/miniforge. To install the latest version on your system (your operating system and architecture will automatically be detected):


curl ‐L ‐O "https://github.com/conda‐forge/miniforge/releases/latest/download/Miniforge3‐$(uname)‐$(uname ‐m).sh"
sh./Miniforge3‐$(uname)‐$(uname ‐m).sh

3 Create and activate a conda environment for Pharokka:


conda create ‐n pharokkaENV ‐c conda‐forge ‐c bioconda pharokka
conda activate pharokkaENV

4 Download Pharokka's databases.


install_databases.py ‐o pharokka_db

#### Run Pharokka

5 Run the example:


pharokka.py ‐i cook_complete_genomes.fasta ‐o cook_pharokka_output ‐t 8 ‐d pharokka_db ‐m

For more detail on Pharokka's specific parameter options, see Table 1.

**Table 1:** pharokka.py Parameters

Parameter	Example usage	Explanation
***Core***		
‐i, --input	‐i genomes.fasta	Input genome(s) in FASTA format (or GenBank, see --genbank below); required
‐o, --output	‐o pharokka_output	Output directory
‐d, --database	‐d pharokka_db	Path to Pharokka database
‐t, --threads	‐t 8	Number of CPU threads to use (defaults to 1)
‐f, --force	‐f	Overwrites existing output
***Auxiliary***		
‐p, --prefix	‐p cook	Prefix for output files. Optional; defaults to “pharokka” if not provided
‐l, --locustag	‐l PHAGE1	User‐specified locus tag for GFF/GBK files; optional; random tag generated if not provided
‐m, --meta	‐m	Enables meta mode for metavirome samples. Improves computational efficiency via parallelization across CPUs
‐s, --split	‐s	Split mode for metavirome samples (requires ‐m); outputs separate FASTA, GFF, and GenBank files per contig
‐g, --gene_predictor	‐g phanotate	Gene predictor to use [options of phanotate, prodigal, prodigal‐gv, pyrodigal‐rv and genbank (used only with --genbank)]; defaults: phanotate (normal), prodigal‐gv (meta mode)
‐c, --coding_table CODING_TABLE	‐c 11	Translation table for Prodigal with ‐g prodigal; default is 11
‐e, --evalue EVALUE	‐e 1e‐5	E‐value threshold for MMseqs2 (PHROGs profiles, VFDB, CARD) and PyHMMER PHROGs searches; default is 1e‐5
--fast, --hmm_only	--fast	Runs only PyHMMER (HMMs) with PHROGs; typically, slow for large metagenome inputs
--custom_hmm	--custom_hmm mydb.h3m	Use a custom HMM database (.h3m), created with create_custom_hmm.py
--mmseqs2_only	--mmseqs2_only	Runs MMseqs2 only (PHROGs, CARD, VFDB); default in meta mode
--meta_hmm	--meta_hmm	Overrides --mmseqs2_only in meta mode to run both MMseqs2 and PyHMMER
--genbank	--genbank	Input file is GenBank instead of FASTA; CDS calls are preserved and re‐annotated
--dnaapler	--dnaapler	Re‐orients contigs to start with terminase large subunit if found using Dnaapler; recommended over --terminase unless you already know the terminase large subunit coordinates
--terminase	--terminase	Terminase‐based re‐orientation flag (single genome input only; requires strand and start options)
--terminase_strand	--terminase_strand pos	Strand of terminase large subunit (pos or neg)
--terminase_start	--terminase_start 12345	Start coordinate of terminase large subunit
--skip_mash	--skip_mash	Skips Mash search against INPHARED
--mash_distance MASH_DISTANCE	--mash_distance 0.2	Mash distance threshold for INPHARED search; default is 0.2
--trna_scan_model {general,bacterial}	--trna_scan_model bacterial	tRNAscan‐SE model to use; defaults to general
--skip_extra_annotations	--skip_extra_annotations	Skips tRNAscan‐SE, MinCED, and Aragorn
--minced_args MINCED_ARGS	--minced_args "--minNR 2 ‐minRL 21"	Specific arguments for MinCED
--sensitivity SENSITIVITY	--sensitivity 9.5	Sets MMseqs2 sensitivity parameter; defaults to 8.5

#### Run Pharokka with custom database (optional)

6 To illustrate the functionality of running Pharokka with user‐specified custom HMM database, first download the example test multiple sequence alignments (MSAs) in FASTA format (Phrog 133, Phrog 142, and Phrog 184). You need these to build the custom HMMs.


mkdir ‐p custom_msas
for f in 133 142 184; do
curl ‐L ‐o custom_msas/phrog_${f}.fasta \ https://raw.githubusercontent.com/gbouras13/pharokka/refs/heads/master/tests/test_data/custom_db/msas/phrog_${f}.fasta
done

7 Then build the HMM profile database using the directory with MSAs as input:


create_custom_hmm.py ‐i custom_msas ‐o custom_hmms ‐p phrogs_133_142_184

  This will create the required database file:


custom_hmms/phrogs_133_142_184.h3m

8 Run Pharokka with custom HMM database:


pharokka.py ‐i cook_complete_genomes.fasta ‐o cook_pharokka_output_custom_hmms ‐t 8 ‐d pharokka_db ‐m --custom_hmm custom_hmms/phrogs_133_142_184.h3m

## Basic Protocol 2 ENHANCED PROKARYOTIC VIRAL METAGENOME PROTEIN ANNOTATION USING PROTEIN STRUCTURES WITH PHOLD

The aim of this protocol is to enhance the sequence homology‐based annotations generated in Basic Protocol 1 with protein structure homology‐based annotations using Phold. The protocol begins by detailing steps on how to install Phold into a conda environment (with specific recommendations based on the user's hardware), followed by running Phold for structure‐based annotation. It then details optionally running Phold on a custom database of user‐provided protein structures.

### Necessary Resources

#### Hardware

A computer with a minimum of 1 CPU core, 16 GB of RAM, and 15 GB of free disc storage for the database install
  It is highly recommended that a GPU with at least 8 GB VRAM is also available. Phold will run without GPU but will be prohibitively slow (the example dataset will take hours).
  Our tests using the example dataset leveraging 8 CPU cores operating at base clock speeds of 2.4 GHz, using 16 GB of RAM combined with a NVIDIA A100 40 GB GPU enabled the completion of the Phold annotation run within 5 min.

##### Software

Linux (or emulators such as Windows Linux Subsystem) or MacOS operating system

##### Files

Test data: the Pharokka output GenBank file of 63 complete metagenome assembled phage genomes generated in Basic Protocol 1

#### Download and installation of software, example data, and required databases

1 Create and activate a conda environment for Phold.
  This protocol details installing Phold in a conda environment in four different scenarios: (a) your machine does not have a GPU; (b) your machine has a modern NVIDIA GPU installed; (c) your machine is manufactured by Apple and has an Apple Silicon chip (e.g., M‐series); and (d) your machine has a modern GPU installed from a different vendor than NVIDIA (e.g., AMD).
  Exact installation commands will depend on the make and model of GPU you have available (and whether you have one at all), as this affects the compatibility with PyTorch. Please choose the installation option below that is suitable for your hardware.
  a. If you have no GPU, the default bioconda install (CPU‐only) will suffice:


conda create ‐n pholdENV ‐c conda‐forge ‐c bioconda phold

  b. If your machine has an NVIDIA GPU, you can create a conda environment with a CUDA‐compatible PyTorch installation in one line automatically:


conda create ‐n pholdENV ‐c conda‐forge ‐c bioconda phold pytorch=*=cuda*

  c. If your Apple machine has an Apple Silicon chip with integrated GPU cores (e.g., M‐series), you can create a conda environment with a GPU compatible PyTorch version as follows by installing from the PyTorch channel:


conda create ‐n pholdENV
conda activate pholdENV
conda install ‐c pytorch pytorch torchvision torchaudio
conda install ‐c conda‐forge ‐c bioconda phold

  d. If you have a GPU from a different vendor (e.g., AMD) or have older hardware (e.g., CUDA drivers), we direct you to follow the instructions at: https://pytorch.org and https://pytorch.org/get‐started/previous‐versions/ to install the appropriate version of PyTorch for your machine using pip. For example, to install Phold on a machine with a modern AMD GPU and ROCM 7.2 installed:


conda create ‐n pholdENV pip
conda activate pholdENV
pip install torch torchvision --index‐url https://download.pytorch.org/whl/rocm7.2
conda install ‐c conda‐forge ‐c bioconda phold

  Once you have created the environment, simply activate it:


conda activate pholdENV

2 Download Phold's databases.

phold install ‐d phold_db

  If you have an NVIDIA GPU, add --foldseek_gpu to ensure compatibility with Foldseek's GPU accelerated prefilter (Kallenborn et al., 2025).
3 (Optional) If you plan to run Phold on many samples, you may wish to find the optimal --batch_size by running Phold with autotune. This will print the optimal batch size for your hardware to the terminal screen.


phold autotune ‐d phold_db

#### Run Phold

4 Run Phold on the example Pharokka GenBank format output file:


phold run ‐i cook_pharokka_output/pharokka.gbk ‐o cook_phold_output ‐t 8 ‐d phold_db

  If your machine has a modern NVIDIA GPU, add --foldseek_gpu to use Foldseek's GPU accelerated prefilter (Kallenborn et al., 2025). If you have run phold autotune to find the optimal batch size, also add this (e.g., if the optimal batch size reported by autotune is 11 add --batch_size 11).
  For more detail on Phold's specific parameter options, see Table 2.

**Table 2:** phold run Parameters

Parameter	Example usage	Explanation
***Core***		
‐i, --input	‐i genome.gbk	Path to input file (GenBank or nucleotide FASTA); required
‐o, --output	‐o phold_output	Output directory
‐d, --database	‐d phold_db	Path to installed phold database
‐t, --threads	‐t 8	Number of CPU threads to use (defaults to 1)
‐f, --force	‐f	Overwrites existing output
***Auxiliary***		
‐p, --prefix	‐p cook	Prefix for output files; optional; defaults to “phold” if not provided
--foldseek_gpu	--foldseek_gpu	Enables Foldseek GPU acceleration
‐e, --evalue	‐e 1e‐3	E‐value threshold for Foldseek; default: 1e‐3
--batch_size	--batch_size 16	Batch size for ProstT5; default: 1
--custom_db TEXT	--custom_db mydb	Path to custom Foldseek database
--cpu	--cpu	Force CPU‐only execution for ProstT5 (no GPU)
--custom_db TEXT	--custom_db mydb	Path to custom Foldseek database
--omit_probs	--omit_probs	Skips 3Di probability output (protein‐level still produced)
--save_per_residue_embeddings	--save_per_residue_embeddings	Saves ProstT5 embeddings per residue to HDF5 file
--save_per_protein_embeddings	--save_per_protein_embeddings	Saves mean ProstT5 embeddings per protein to HDF5 file
--mask_threshold FLOAT	--mask_threshold 25	Masks 3Di residues below this confidence threshold for Foldseek; default: 25
--finetune	--finetune	Uses finetuned ProstT5Phold encoder + CNN trained on phage proteins
--vanilla	--vanilla	Uses vanilla CNN model (CASP14‐trained) with ProstT5Phold encoder
--hyps	--hyps	Annotates only hypothetical proteins from a Pharokka GenBank input
--autotune	--autotune	Automatically determines optimal batch size for hardware; best for large datasets
‐s, --sensitivity	‐s 9.5	Foldseek sensitivity parameter; default: 9.5
--keep_tmp_files	--keep_tmp_files	Retains temporary files (e.g., the full foldseek_results.tsv).
--card_vfdb_evalue	--card_vfdb_evalue 1e‐10	Stricter E‐value threshold for CARD and VFDB Foldseek hits; default: 1e‐10
--separate	--separate	Outputs separate GenBank files per contig
--max_seqs	--max_seqs 10000	Max results per query after prefilter; default: 1000
--ultra_sensitive	--ultra_sensitive	Maximum sensitivity mode (skips Foldseek prefilter); not recommended for large datasets
--extra_foldseek_params TEXT	--extra_foldseek_params "‐c 0.5"	Additional parameters for Foldseek search
--restart	--restart	Restarts pipeline from Foldseek output processing step

#### Run Phold with custom structure database (optional)

5 To illustrate the functionality of running phold with a user‐specified custom Foldseek database of protein structures, first download the example test structures in PDB format.


mkdir ‐p custom_pdbs
for f in \
k149_60443_CDS_0081 \
phage_comp_44_cycle_1_CDS_0103 \
phage_comp_9_cycle_1_CDS_0042
do
curl ‐L ‐o custom_pdbs/${f}.pdb \ https://raw.githubusercontent.com/gbouras13/phold/main/tests/test_data/protocols_pdbs/${f}.pdb
done

6 Then build the custom Foldseek database using the directory with protein structures as input.


mkdir ‐p custom_db
foldseek createdb custom_pdbs custom_db/db

  If you have an NVIDIA GPU, to ensure you can use --foldseek_gpu with Phold, please also run:


foldseek makepaddedseqdb custom_db/db custom_db/db_pad

7 Run Phold with custom structures:


phold run ‐i cook_pharokka_output/pharokka.gbk ‐o cook_phold_output_custom_structures ‐t 8 ‐d phold_db ‐f --custom_db custom_db/db

  If you have an NVIDIA GPU, you will need to use the padded database along with --foldseek_gpu.


phold run ‐i cook_pharokka_output/pharokka.gbk ‐o cook_phold_output ‐t 8 ‐d phold_db ‐f --custom_db custom_db/db_pad --foldseek_gpu (Table 2)

## Basic Protocol 3 FURTHER PROKARYOTIC VIRAL METAGENOME PROTEIN USING GENOME SYNTENY AND PROTEIN LANGUAGE MODELS WITH PHYNTENY

The aim of this protocol is to enhance the sequence homology‐based annotations generated in Basic Protocol 1 augmented with structure homology‐based annotations generated in Basic Protocol 2 with genome synteny and protein language model‐based annotations using Phynteny. The protocol begins by detailing steps on how to install Phynteny into a conda environment (with specific recommendations based on the user's hardware), followed by running Phynteny.

### Necessary Resources

#### Hardware

A computer with a minimum of 1 CPU core, 8 GB of RAM, and 1 GB of free disc storage for the database install
  It is highly recommended that a GPU with at least 8 GB VRAM is also available. Phynteny will run without GPU but will be prohibitively slow (the example dataset will take hours).
  Our tests using the example dataset leveraging 1 CPU core operating at base clock speeds of 2.4 GHz, using 16 GB of RAM combined with a NVIDIA A100 40 GB GPU enabled the completion of Phynteny within 3 min.

##### Software

Linux (or emulators such as Windows Linux Subsystem) or MacOS operating system

##### Files

Test data: the Phold output GenBank file of 63 complete metagenome assembled phage genomes from human gut samples generated in Basic Protocol 2

#### Download and installation of software and required databases

1 Create and activate a conda environment for Phynteny.
  Much like Basic Protocol 2, this protocol details installing Phynteny in four different scenarios: (a) your machine does not have a GPU; (b) your machine has a modern NVIDIA GPU installed; (c) your machine is manufactured by Apple and has an Apple Silicon chip; and (d) your machine has a modern GPU installed from a different vendor (e.g., AMD).
  Exact installation commands will depend on the make and model of GPU you have available (and whether you have one at all), as this affects the compatibility with PyTorch. Please choose the installation option below that is suitable for your hardware.
  a. If you have no GPU, the default bioconda install (CPU‐only) will suffice:


conda create ‐n phyntenyENV ‐c conda‐forge ‐c bioconda phynteny_transformer

  b. If your machine has an NVIDIA GPU, you can create a conda environment with a CUDA‐compatible PyTorch installation in one line automatically:


conda create ‐n phyntenyENV ‐c conda‐forge ‐c bioconda phynteny_transformer pytorch=*=cuda*

  c. If your machine has an Apple Silicon chip with integrated GPU cores (e.g., M‐series), you can create a conda environment with a GPU compatible PyTorch version as follows by installing from the PyTorch channel:


conda create ‐n phyntenyENV
conda activate phyntenyENV
conda install ‐c pytorch torchvision torchaudio
conda install ‐c conda‐forge ‐c bioconda phynteny_transformer

  d. If you have a GPU from a different vendor (e.g., AMD) or have older hardware (e.g., CUDA drivers), we direct you to follow the instructions at: https://pytorch.org and https://pytorch.org/get‐started/previous‐versions/ to install the appropriate version of PyTorch for your machine using pip. For example, to install Phynteny Transformer on a machine with an AMD GPU and ROCM 7.2:


conda create ‐n phyntenyENV pip
conda activate phyntenyENV
pip install torch torchvision --index‐url https://download.pytorch.org/whl/rocm7.2
conda install ‐c conda‐forge ‐c bioconda phynteny_transformer

  Once you have created the environment, simply activate it:


conda activate phyntenyENV

2 Download Phynteny Transformer's databases:


install_models ‐o phynteny_transformer_db

#### Run Phynteny

3 Run Phynteny Transformer:


phynteny_transformer cook_phold_output/phold.gbk ‐o cook_phynteny_output ‐m phynteny_transformer_db/models/ (Table 3)

  For more detail on Phyntney's specific parameter options, see Table 3.

**Table 3:** Phynteny Parameters

Parameter	Example usage	Explanation
***Core***		
INPUT	genome.gbk	Path to input file (GenBank or nucleotide FASTA); required
‐o, --output	‐o phynteny_output	Output directory
‐m, --models	‐m /path/to/models	Path to downloaded phynteny models
‐f, --force	‐f	Overwrites existing output
***Auxiliary***		
‐p, --prefix	‐p cook	Prefix for output files; optional; defaults to “phynteny” if not provided
--confidence‐threshold	‐c 0.8	The confidence threshold required to assign a predicted function to an unannotated gene; default 0.8
‐b, --batch_size	--batch_size 32	The batch size to use during Phynteny inference
--save_attention_weights	--save_attention_weights	Saves the transformer's attention matrices to an output file; useful for visualizing which surrounding genes influenced a specific functional prediction

## Basic Protocol 4 VISUALIZATION OF PROKARYOTIC VIRAL METAGENOME ANNOTATIONS WITH PHOLD PLOT WEB ASSEMBLY APPLICATION

The aim of this protocol is to show how the Phold Plot Web Assembly Application can be used for visualizing the prokaryotic virus genomic annotations generated in Basic Protocols 1 to 3.

### Necessary Resources

#### Hardware

A computer with a minimum of 1 CPU core and 8 GB of RAM
  Our tests using the example dataset leveraging 1 CPU core on a MacBook Pro M1 (2021) took <1 min to generate the two plots shown.

##### Software

Any operating system may be used along with any modern web browser

##### Files

Test data: the Phynteny output GenBank file generated in Basic Protocol 3

**Figure 1:**
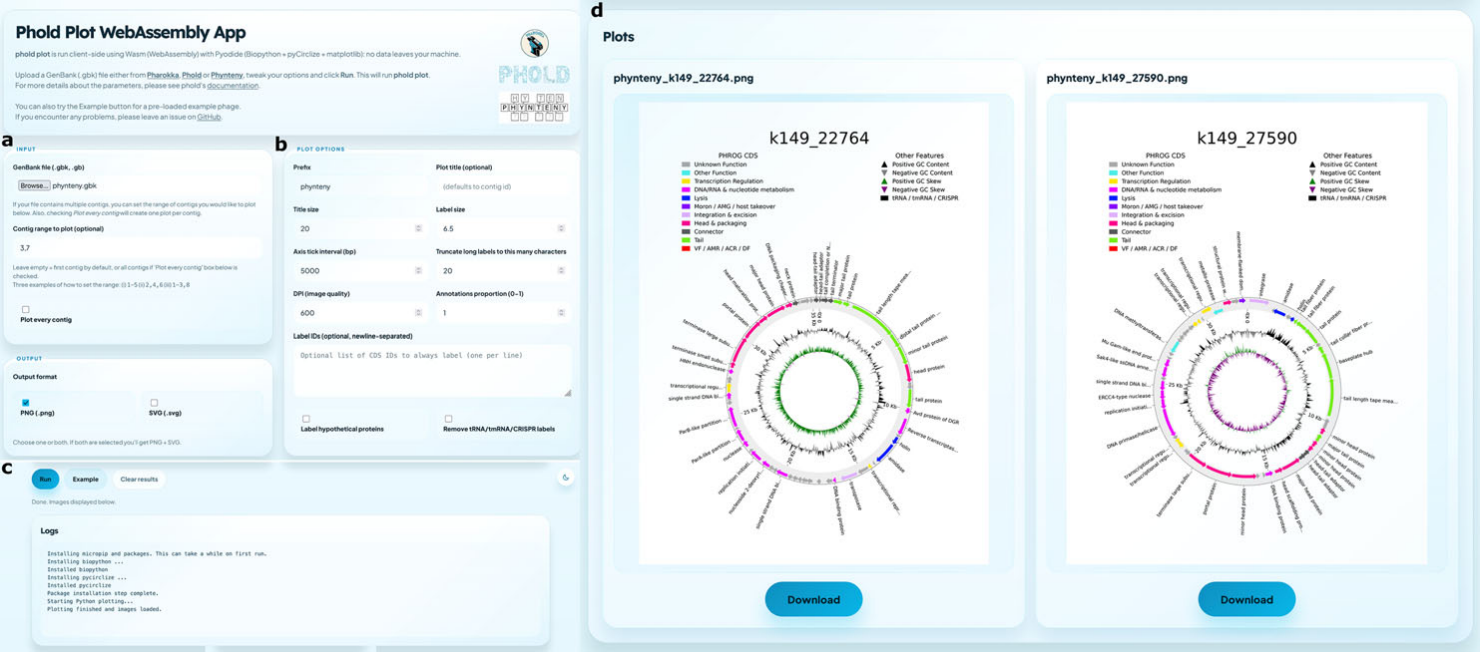
Phold Plot Web Assembly Application website with (**A**) upload box, (**B**) parameter plot options, (**C**) run box, and (**D**) download box for the plots of interest.

1 Open the Phold Plot Web Assembly Application at: https://gbouras13.github.io/phold‐plot‐wasm‐app/ in a modern web browser (e.g., Google Chrome, Mozilla Firefox, Safari, Microsoft Edge, etc.).
2 Upload your GenBank output by clicking the Choose File button; in this case, the specific file of interest is cook_phynteny_output/phynteny.gbk as output from Basic Protocol 3. Once uploaded, this button becomes “Browse” (as depicted in Fig. 1A).

3 Choose the contigs of interest to plot by specifying the contig range. In Figure 1A, contigs 3 and 7 are specified as an example. If you would like to plot all contigs, alternatively click the “Plot every contig” box.
  Caution: This might take a long time for large datasets and be visually overwhelming.
4 Choose your specific plotting parameters (Fig. 1B).
5 Click Run (Fig. 1C) to generate and then click Download (Fig. 1D) to download the desired genomic plots.

## COMMENTARY

### Background Information

Pharokka, Phold, and Phynteny were designed to provide consistent, comprehensive annotation of phage genomes, scaling efficiently to handle metagenome‐scale viral datasets. Each software program was designed to accept the output of the previous tool as input to iteratively propagate and improve annotations using different information sources and bioinformatics algorithms. Pharokka undertakes genome feature identification (gene calling, tRNA, tmRNA, and CRISPR repeat detection), combined with traditional sequence‐homology‐based protein annotation and taxonomic assignment to closely related phages in the INPHARED database (Cook et al., 2021). Phold extends protein annotations utilizing the ProstT5 protein language model (Heinzinger et al., 2024) to predict Foldseek 3Di structural tokens and uses Foldseek (van Kempen et al., 2024) to conduct structure‐homology searches against a database of 1.36 M phage protein structures. Phynteny then uses the ESM2 protein language model (Lin et al., 2023) along with genome synteny information to attempt to assign remaining unknown proteins one of nine broad PHROG (Prokaryotic Virus Remote Homologous Groups) categories: “head and packaging”, “connector”, “tail”, “DNA, RNA and nucleotide metabolism”, “integration and excision”, “lysis”, “transcription regulation”, “moron, auxiliary metabolic gene and host takeover”, or “other” (Terzian et al., 2021).

### Critical Parameters

Care should be taken to select the gene predictor for Pharokka using ‐g. By default, for metagenome datasets with multiple contigs, Pharokka will use prodigal‐gv (Camargo et al., 2024; Hyatt et al., 2010; Larralde, 2022) as it supports detecting some alternative coding tables and is extremely fast. However, users interested in capturing smaller ORFs should consider using Phanotate (McNair et al., 2019) (‐g phanotate) as this gene caller generally detects more smallORFs than prodigal, prodigal‐gv or prodigal‐rv. If users believe RNA phages are a considerable portion of their dataset, pyrodigal‐rv should be considered as it specifically supports translation tables for RNA viruses. As discussed in more detail in the Troubleshooting section, if the CDS density is low for your dataset, this indicates the gene caller is not appropriate, and a different one might yield better results. Additionally, we recommend Dnaapler (Bouras et al., 2024) via the --dnaapler parameter if users would like to consistently reorient their phages to begin with the terminase large subunit, which may be especially useful for syntenic or variant analyses.

For Phold, we strongly recommend --foldseek_gpu if the user has an NVIDIA GPU available. GPU‐based prefilter acceleration speeds up the Foldseek search step by 2‐ to 3‐fold (Kallenborn et al., 2025). We also recommend considering using phold autotune to find the maximum allowable --batch_size if the user has a large dataset to annotate, as the ProstT5 3Di inference step will be much faster than the default --batch_size 1. If maximum recall is of the utmost importance for the user (with the trade‐off of heavy resource usage), we recommend increasing --max_seqs from its default of 1000 to 20000 or 50000, as this will marginally increase Phold's annotation rate by allowing more hits through Foldseek's prefilter.

When running Phynteny, the confidence score (‐c or ‐confidence) dictates the stringency of functional assignments. The default setting of ‐c 0.8 balances precision and recall; users requiring more conservative functional predictions may wish to increase this threshold, whereas those aiming for maximum exploratory coverage can lower it. Furthermore, if interpretability is a priority, users can use the --save_attention_weights flag to extract and save the transformer model's attention matrices, allowing users to visualize specifically which surrounding genes in the local syntenic neighborhood most heavily influenced the functional prediction of a given unannotated protein.

For the Phold Plot Web Assembly application, the primary parameter that affects the plotting is the “annotations proportion”. For larger genomes (e.g., >100,000 nucleotides), this may need to be reduced from 1 to 0.5, otherwise the visualization will become too crowded to be easily interpreted and annotation labels may overlap.

### Troubleshooting

See Table 4 for a troubleshooting guide for annotation of viral metagenomes with Pharokka, Phold and Phynteny.

**Table 4:** Troubleshooting Guide for Annotation of Viral Metagenomes With Pharokka, Phold, and Phynteny

Problem	Possible cause	Solution
Pharokka, Phold, or Phynteny crashes, crashes with an out‐of‐memory error, hangs during execution, takes many hr, or exceeds job run limits	Input file is not a FASTA, or the input FASTA file is suboptimally large (e.g., many thousands of contigs)	Check input is in FASTA format; if the input FASTA is very large, split input into smaller chunks (tens or hundreds of contigs) e.g., using seqkit split
Gene calling fails or hangs in Pharokka	Input FASTA file contains contigs that are too short (<1000 or >1 M nucleotides)	Remove these contigs from your input, as they are unlikely to represent phage genomes
Pharokka, Phold, or Phynteny fails or gives unexpected output	Input FASTA headers longer than 40 characters or contains special characters (including “:”, “∼”, and “;”), spaces	Rename offending headers to shorten them, remove special characters and spaces
Phold not predicting 3Di tokens for large (>5000 amino acid) proteins	Out of memory errors on your GPU	Use a GPU with larger VRAM or our Google Colab notebook
Web assembly Phold Plot application freezes or hangs	Input GenBank file is too large, or plots are requested for too many contigs	Reduce the Annotations proportion parameter for large genomes (e.g., >100,000 nucleotides in length), limit plotting to only a selection (e.g., <5) of contigs at a time

On extremely large jobs (e.g., thousands of genomes), Pharokka, Phold, or Phynteny may crash due to out‐of‐memory errors, hang during execution, or fail to complete within the allocated time (if run via cloud or HPC). The --restart parameter may be used to restart Phold from an intermediate checkpoint with larger resource and time specifications. Alternatively, if extremely large, we recommend splitting the input genome into chunks with e.g., Seqkit (Shen et al., 2024) using seqkit split, then running Pharokka, Phold, and Phynteny in parallel across multiple input chunks.

The input file of genome(s) needs to be checked to ensure it is in standards‐compliant FASTA format. Extremely short (<1000 nucleotides) or extremely long (>1 M nucleotides) contigs that are unlikely to represent phage genomes should be removed, as gene calling may fail. In cases where Pharokka hangs on the Phanotate step, this is usually where the input contig is extremely large (>1 M nucleotides) and unlikely to be a phage genome.

If Pharokka, Phold, or Phynteny gives unexpected output or fails outright, please check the headers of your input FASTA. While care has been taken to ensure the tools can handle unusual headers, edge cases likely remain. Special characters (especially “:”, “∼”, and “;”), spaces in headers and extremely long headers (>40 characters in length) may cause downstream issues. We recommend naming any FASTA headers that do not conform to those requirements.

Phold may not be able to predict Foldseek 3Di tokens for large (>5000 amino acids) proteins, especially if your GPU has limited VRAM. Phold should warn gracefully, with an annotation for these proteins skipped. If these proteins are important, we recommend either rerunning Phold on a GPU with larger VRAM or using our Google Colab notebook (https://colab.research.google.com/github/gbouras13/phold/blob/main/run_pharokka_and_phold_and_phynteny.ipynb) to access larger GPUs.

We encourage users to submit any bugs or issues through the GitHub repositories to track the development of fixes at: https://github.com/gbouras13/pharokka/issues; https://github.com/gbouras13/phold/issues; and https://github.com/susiegriggo/Phynteny_transformer/issues.

### Understanding Results

The primary interpretable outputs of the three tools are the .csv and .tsv files detailing the annotations in detail. Each tool also outputs a standards‐compliant GenBank (.gbk format) annotation file, which can be uploaded to International Nucleotide Sequence Database Collaboration (INSDC) databases (Karsch‐Mizrachi et al., 2025). We recommend suvtk (https://github.com/LanderDC/suvtk) to aid this process, although we do not detail its use in this protocol. Pharokka and Phold output additional annotation outputs such as protein calls in nucleotide and amino acid format, alternative INSDC compliant formats (e.g., GFF3) and specific antimicrobial resistance genes and virulence factor annotations. We direct users to the Pharokka documentation (https://pharokka.readthedocs.io/en/latest/output/) and the Phold documentation (https://phold.readthedocs.io/en/latest/output/) for more details.

Main Pharokka outputs of interest include:

(1) pharokka_length_gc_cds_density.tsv, which gives the input length, GC content, predicted translation table and CDS density per contig. As a heuristic, if the contig CDS density is <80%, this indicates suboptimal gene calling and therefore the contig likely utilizes an alternative translation table.
(2) pharokka_top_hits_mash_inphared.tsv, which gives the INPHARED genome found with the smallest Mash distance for each input contig (if there are any hits below the Mash distance threshold, default 0.2).
(3) pharokka_cds_functions.tsv, which gives summary per‐contig counts of CDS, tRNAs, tmRNAs, CRISPR repeats, virulence factors annotated, antimicrobial resistance genes (AMR) annotated, and counts of CDS belonging to each of the nine PHROG functional categories, along with counts of the remaining unknown function proteins.
(4) pharokka_cds_final_merged_output.tsv, which gives detailed per CDS annotation statistics, including MMseqs2 and/or PyHMMER alignment bitscores and E‐values, sequence identities and the exact PHROG group the top hit target belongs to. The specific PHROG group can then be analyzed in more detail using the webserver at: https://phrogs.lmge.uca.fr.

Main Phold outputs of interest include:

(1) phold_all_cds_functions.tsv, which is much like pharokka_cds_functions.tsv and gives summary per‐contig counts of CDS, virulence factors, antimicrobial resistance genes (AMR), anti‐phage defense genes, anti‐CRISPR genes and toxin‐antitoxin genes annotated, and counts of CDS belonging to each of the nine PHROG functional categories, along with counts of the remaining unknown function proteins.
(2) phold_per_cds_predictions.tsv, which is much like pharokka_cds_final_merged_output.tsv, giving detailed per CDS annotation information. Of particular interest is the tenth column annotation_confidence, which gives a High, Medium, or Low heuristic category for annotation quality. More information on how these thresholds are determined can be found at: https://phold.readthedocs.io/en/latest/output/.

The main Phynteny output of interest is phynteny.tsv, which gives updated functional annotations for each protein in column seven phynteny_category. Column nine phynteny_confidence gives the Phynteny confidence score (ranging from 0 to 1): the higher the score, the more accurate the model prediction. Generally, scores >0.8 indicate a high degree of confidence in the updated annotation.

**Figure 2:**
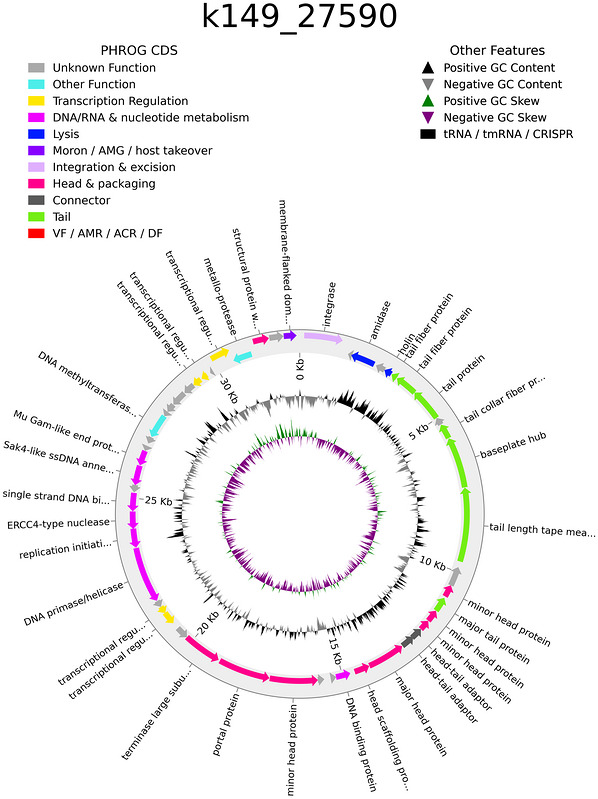
Example phage genomic map visualization from Basic Protocol 4 (specifically, contig 3 from the example dataset generated).

The genomic map plots generated in Basic Protocol 4 (see Fig. 2 for an example) contain key information in concentric rings (n.b., phage genomes are not necessarily circular, but the plot functionality will show them as circular for ease of visualization). Each CDS is arranged on the outermost ring as arrows, with clockwise arrows indicating positive‐strand CDS and counterclockwise negative‐strand. CDS are colored by their PHROG functional category, with the legend mapping color to category shown at the top left of Figure 2, and annotated CDS have their specific functional annotations labeled in text. Unannotated CDS will be colored grey. The inner two circles are GC content and GC skew. The GC skew should be correlated with the likely strandedness of CDS in this region, with regions of positive skew indicating positive strand CDS and vice versa, as is observed in Figure 2. The GC content shows the deviation from the overall genome‐wide average across a sliding window around each nucleotide and indicates regions of unusually high and low GC content.

### Time Considerations

Both Phold and Phynteny scale approximately linearly in input genome count and wall clock time. For enormous datasets (e.g., many thousands of genomes), as each input genome is independently annotated, we recommend splitting the input FASTA into chunks of tens or hundreds of genomes and running Pharokka, Phold, and Phynteny in parallel. Pharokka scales sub linearly with input genome count and wall clock time, due to the way the PHROGs profile database (38,880 sequence profiles) is used as a fixed query database, and the set of input genome proteins is used as the target. Because MMseqs2 profile search prefilters hits, only the top (by default) 1000 hits will pass the prefilter for each input profile. Therefore, for enormous inputs, Pharokka annotation rates may be degraded. This is an additional reason we recommend splitting large datasets into smaller chunks.

### Author Contributions

**George Bouras**: Conceptualization; data curation; formal analysis; writing—original and draft; writing—review and editing. **Susanna Grigson**: Formal analysis; software; validation; writing—original and draft; writing—review and editing. **Lindsay Durr**: Formal analysis; validation; writing—original and draft; writing—review and editing. **Bhavya Papudeshi**: Formal analysis; software; writing—original and draft; writing—review and editing. **Vijini Mallawaarachchi**: Formal analysis; software; writing—original and draft; writing—review and editing. **Sarah Vreugde**: Funding acquisition; project administration; supervision; writing—original and draft; writing—review and editing. **Robert Edwards**: Funding acquisition; project administration; supervision; writing—original and draft; writing—review and editing.

### Conflict of Interest

The authors have no conflicts of interest to declare.

## Data Availability

The data used in this study are all publicly available. The input data required to run these protocols are available at: https://github.com/gbouras13/pharokka and https://github.com/gbouras13/phold.
